# Longitudinal brain-age predictions comprising long-duration spaceflight missions

**DOI:** 10.1038/s41526-026-00575-3

**Published:** 2026-02-18

**Authors:** Ge Tang, Kaustubh R. Patil, Felix Hoffstaedter, Shammi More, Simon B. Eickhoff, Steven Jillings, Ben Jeurissen, Elena Tomilovskaya, Darius Gerlach, Inna Nosikova, Alexandra Riabova, Ekaterina Pechenkova, Viktor Petrovichev, Ilya Rukavishnikov, Lyudmila Makovskaya, Angelique Van Ombergen, Floris L. Wuyts, Peter zu Eulenburg

**Affiliations:** 1 Institute for Neuroradiology, University Hospital, LMU Munich, Munich, Germany; 2 Graduate School of Systemic Neurosciences, LMU Munich, Munich, Germany; 3 Institute of Neuroscience and Medicine, Brain & Behaviour (INM-7), Research Centre Jülich, Jülich, Germany; 4 Institute of Systems Neuroscience, Medical Faculty, Heinrich Heine University Düsseldorf, Düsseldorf, Germany; 5 Lab for Equilibrium Investigations and Aerospace, University of Antwerp, Antwerp, Belgium; 6 Imec/Vision Lab, University of Antwerp, Antwerp, Belgium; 7 SSC RF—Institute of Biomedical Problems, Russian Academy of Sciences, Moscow, Russia; 8 Institute of Aerospace Medicine, German Aerospace Center (DLR), Cologne, Germany; 9 Laboratory for Cognitive Research, HSE University, Moscow, Russia; 10 Radiology Department, Federal Center of Treatment and Rehabilitation, Moscow, Russia; 11 Radiology Department at the Medical Research and Educational Center, Lomonosov Moscow State University (MSU), Moscow, Russia; 12 Department of Translational Neurosciences—ENT, University of Antwerp, Antwerp, Belgium; 13 Directorate of Human and Robotic Exploration, European Space Agency (ESA), Noordwijk, Netherlands; 14 German Center for Vertigo and Balance Disorders, University Hospital, LMU Munich, Munich, Germany

**Keywords:** Neuroscience, Physiology, Medical research, Neurology

## Abstract

Our study investigates the effects of long-duration spaceflight on brain aging in spacefarers using structural MRI and machine learning models. Pre-, post-, and follow-up scans of ROS cosmonauts ESA astronauts, and matched Earth-bounding controls were analyzed. We found a considerable difference between the spacefareres and the control group, especially in the ESA cohorts (ß = 0.63). In the ROS cohorts, we observed a difference between the pre- and post-flight scans. A post-hoc analysis revealed that the pre-flight brain age delta was 0.842 years less than the immediate post-flight brain age delta after long-duration spaceflight. All three machine learning models showed good to excellent intraclass correlation coefficients (ICC) between the two consecutive MRI sessions. Our findings suggest that long-duration spaceflight may have an effect on human brain aging as observed from MRI.

## Introduction

Living in space for up to a year is now an ever more common feat for space travellers through modern technology. This accomplishment for humans, however, is not achieved without physiological impacts. Recent findings in space medicine research indicate potential adverse effects of long-duration spaceflight (LDSF) on the brain’s structural integrity^1^. Key observations include an expanded cerebrospinal fluid (CSF) compartment, enlarged perivascular spaces, a reduction in cortical grey matter (GM) volume, increased levels of brain-structure proteins in peripheral blood, and spaceflight associated neuro-ocular syndrome (SANS) after landing back on Earth^1–5^. All of which are pointing to the potential for brain abnormality and persisting brain-structural changes due to prolonged exposure to LDSF. These alterations of human brain-structural integrity were all observed via neuroimaging up to six months after return from a mission of similar length and do not show a trajectory for a full return to the preflight baseline.

These discoveries have now led to the motivation on our part to observe the aging processes in those space travellers and to delineate a comprehensive parameter to quantify the overall impact of prolonged exposure to LDSF on the human brain and its grey matter. A promising approach in this regard might be “brain age” prediction from whole-brain magnetic resonance imaging (MRI). By comparing predicted brain age to actual chronological age, one could observe and scale alterations in the brain due to time spent in space. In recent years, unmatched or accelerated brain aging has demonstrated to be associated with neurodegenerative diseases^6^, mental disorders^7^ and other health conditions^8,9^. Large dataset-based machine learning models have just now opened a door for predicting brain age from structural MRI with good precision on an individual level^10^, thus, making the method applicable to our research question.

In the realm of machine-learning-based brain-age models, there are currently two predominant classes of models: traditional machine learning and deep learning based models^8^. Our study made use of three different state-of-the-art brain-age models that use T1-weighed MRI data as input to align with our unique dataset. This set includes two deep learning models: MCCQRNN, which adjusts for uncertainty during age prediction, and CNN3. Along with a state-of-the-art traditional machine learning model, S4_R4 + GPR, selected from a pool of 128 tested architectures^11–13^. Our dataset stems from an ongoing 10-year-long observational project, which recruited two cohorts of spacefarers along with age-, gender-, and education-matched control subjects. One is a Russian cosmonaut cohort (ROS), and the other is a European astronaut (ESA) cohort.

In this exploratory, methods-focused study, we aimed to investigate the longitudinal brain aging trajectory among spacefarers before, directly after 6-month missions aboard the ISS, and after a follow-up period of half a year, in comparison with the trajectories observed in Earth-bound matched control subjects over the same periods of time. At a time when brain-age prediction methods are used on a wider spectrum than ever, we also wanted to provide a methodological assessment of both the validity and test-retest reliability of the proposed brain-age models using repeated measurements. This study demonstrates feasibility and provides preliminary evidence as a foundation for future work examining spaceflight effects on brain aging.

## Results

### Reliability analysis

**Fig. 1:**
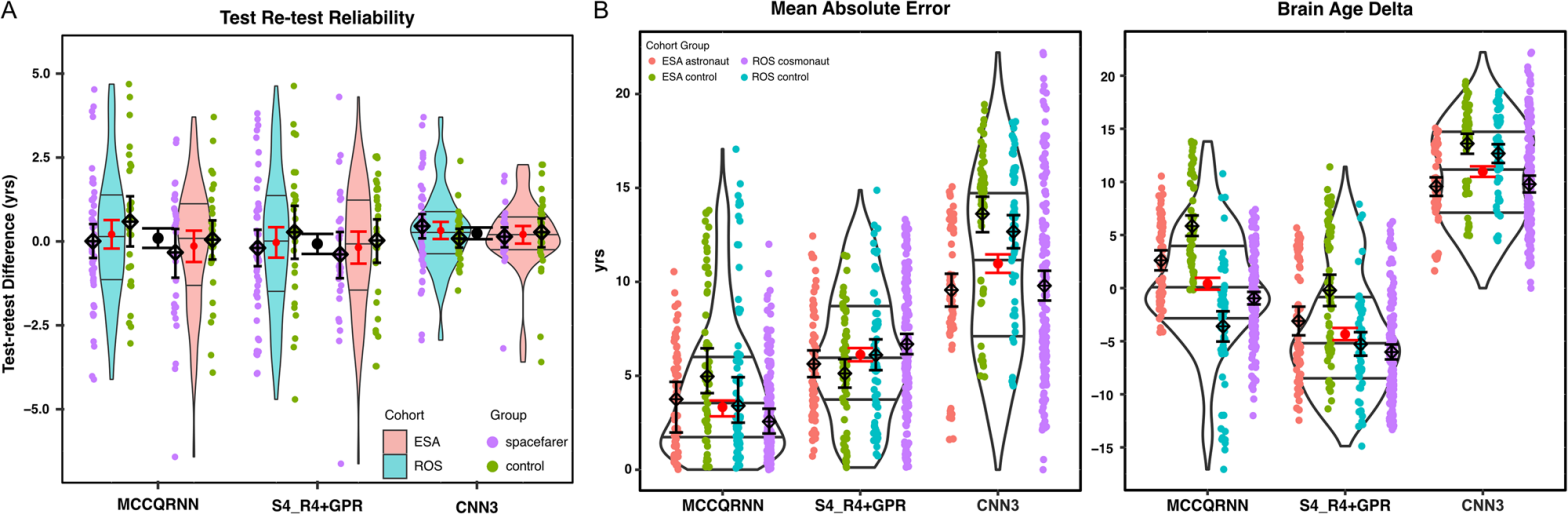
Reliability and validity metrics for brain age prediction models. **A** Assessment of model reliability: Depicts the variation in consecutive MRI scans taken 30 min apart, highlighting the intraclass correlation coefficients (ICC) for both spacefarers and control groups. The red error bars represent the aggregated variation from the pooled cohort that underwent scanning on the same MRI scanner, indicating the consistency of scan results. **B** Model validation: Features violin plots visualizing the mean absolute error (or median absolute error from MCCQRNN model) (MAE) (left panel), and Brain Age Delta (right panel) in predictions from three different machine learning (ML) models. This comparison aims to illustrate the reliability and validity of age prediction performance across the models.

To evaluate measurement precision, we assessed test-retest reliability using MRI scans acquired half an hour apart from the same individuals, a design that eliminates biological variability and provides a stringent test of model stability. All three machine learning models demonstrated excellent reliability (Table 1, with an illustration in Fig. 1A), with intraclass correlation coefficient, ICC(3,1), values of 0.94 (95% CI: 0.92–0.96) for MCCQRNN, 0.94 (95% CI: 0.92–0.95) for S4_R4 + GPR, and 0.97 (95% CI: 0.96–0.98) for CNN3. These values indicate that 94–97% of the prediction variance reflects true individual differences rather than measurement error, exceeding the ICC threshold of 0.90 for clinical applications^14^. The Standard Error of Measurement (SEM) was 1.35, 1.38, and 0.81 years, respectively, indicating that individual measurements typically deviate by less than 1.4 years from the true brain age due to random error. The corresponding minimal detectable change thresholds (MDC95) of 3.73, 3.81, and 2.24 years establish that brain age changes exceeding these values can be confidently attributed to genuine biological processes with 95% confidence. Analysis of individual test-retest differences revealed that only 7% of scans (*n* = 12) exceeded the MDC95 for MCCQRNN, consistent with expected statistical variation, while no scans exceeded this threshold for the other two models. Subgroup analysis demonstrated that good to excellent reliability was maintained across controls and spacefarers (ICC > 0.86 for all models), with consistently low measurement error (SEM < 1.53 y) and narrow change detection thresholds (MDC95 < 4.25 y). These results indicate robust measurement precision of three machine learning models, which is suitable for longitudinal monitoring and intervention studies.

**Table 1:** Evaluation of test-retest reliability and validity metrics for brain age prediction models

			ROS cosmonauts		ESA astronauts		Total(356/163)	Reported
			Controls (66/28)*	Cosmonauts (163/73)	Controls (63/31)	Astronauts (64/31)		
MCCQRNN	Reliability metrics	ICC	0.94(0.87–0.97)	0.88(0.81–0.92)	0.91(0.83–0.96)	0.86(0.72–0.93)	0.94(0.92–0.96)	
		MD(yrs)	0.59(-0.21 — 1.39)	0.11(-0.32 — 0.54)	0.05(-0.57 — 0.67)	–0.33(–1.10 to 0.44)	0.1(–0.19 to 0.39)	
		SD(yrs)	2.06	1.84	1.68	2.10	1.90	
		SEM(yrs)	1.45	1.30	1.19	1.49	1.35	
		MDC95(yrs)	4.03	3.59	3.29	4.12	3.73	
	Validity metrics	MAE(yrs)	5.04(3.76–6.84)	3.00(2.54–3.58)	5.79(4.61–7.41)	3.64(2.78–4.54)	4.02(3.56–4.56)	
		Brain Age Delta(yrs)	–3.60(–5.07 to –2.13)	–0.94(–1.54 to –0.34)	5.86(4.86–6.86)	2.62(1.65–3.59)	0.41(–0.15 to 0.97)	
		Median Absolute Error(yrs)	3.41[1.60, 6.49]	2.56[1.17, 4.48]	4.96[3.02, 8.82]	3.76[1.27, 5.88]	3.33[1.44, 5.76]	2.94(0.22)**
		age-bias slope	–0.05	–0.15	–1.02	–0.53	–0.35	
S4_R4 + GPR	Reliability metrics	ICC	0.88(0.76–0.94)	0.91(0.85–0.94)	0.96(0.91–0.98)	0.94(0.88–0.97)	0.94(0.92–0.95)	
		MD(yrs)	0.28(–0.56 to1.12)	–0.12(–0.56 to 0.32)	0.03(–0.66 to 0.72)	–0.39(–1.12 to 0.34)	–0.08(–0.38 — 0.22)	
		SD(yrs)	2.17	1.89	1.87	1.98	1.95	
		SEM(yrs)	1.53	1.33	1.33	1.4	1.38	
		MDC95(yrs)	4.25	3.70	3.67	3.89	3.81	
	Validity metrics	MAE(yrs)	6.08(5.09–7.21)	6.54(5.70–7.26)	5.00(3.88–6.07)	5.69(4.77–6.61)	6.03(5.54 — 6.53)	4.04–5.2***
		Brain Age Delta(yrs)	–5.28(–6.42 to –4.14)	–6.02(–6.73 to –5.31)	–0.21(–1.73 to 1.31)	–3.10(–4.49 to –1.71)	–4.33(–4.9 to 3.76)	
		Median Absolute Error(yrs)	5.62[3.49, 7.84]	6.3[4.21, 9.59]	5.33[2.30, 7.27]	5.28[3.41, 7.79]	5.7[3.61, 8.57]	
		age-bias slope	–0.05	–0.16	–1.1	–1.19	–0.42	
CNN3	Reliability metrics	ICC	0.98(0.96–0.99)	0.96(0.94–0.98)	0.95(0.90–0.98)	0.97(0.95 — 0.99)	0.97(0.96–0.98)	
		MD(yrs)	0.09(-0.21 — 0.39)	0.33(0.02 — 0.64)	0.28(–0.18 to 0.74)	0.14(–0.17 to 0.45)	0.24(0.06 — 0.42)	
		SD(yrs)	0.77	1.32	1.24	0.86	1.14	
		SEM(yrs)	0.54	0.94	0.88	0.61	0.81	
		MDC95(yrs)	1.51	2.59	2.44	1.68	2.24	
	Validity metrics	MAE(yrs)	12.9(11.6– 13.9)	9.89(8.81–11.0)	13.6(12.0–14.7)	9.59(8.35–10.7)	11.1(10.3–11.7)	2.67
		Brain Age Delta(yrs)	12.7(11.8–13.6)	9.79(8.99–10.6)	13.6(12.6–14.6)	9.57(8.67–10.5)	11.0(10.5–11.5)	
		Median Absolute Error(yrs)	12.9[11.2, 15.7]	8.87[5.88, 13.2]	14.8[13.1, 15.8]	9.59[7.67, 12.9]	11.3[7.21, 14.8]	
		age-bias slope	–0.30	–0.50	–0.90	–0.83	–0.54	

This table presents reliability and validity metrics for three machine learning models applied to brain age prediction, evaluated using two MRI scans acquired within the same session (30-min interval between scans). Reliability Metrics (reflecting reproducibility and measurement consistency): Intraclass Correlation Coefficient, ICC(3,1), with 95% confidence intervals (CI), Mean Difference (MD) with 95% CI, Standard Error of Mean difference (SD), Standard Error of Measurement (SEM), and Minimal Detectable Change at 95% confidence (MDC95). ICC values indicate good to excellent test-retest reliability across all models and cohorts. Low SEM and MDC95 values support the precision of repeated measurements. Validity Metrics (reflecting accuracy and systematic error): Mean Absolute Error (MAE) with 95% CI, Brain Age Delta (mean difference between predicted and chronological age) with 95% CI, Median Absolute Error (Median AE) with interquartile range (IQR), and age-bias slope (delta vs. chronological age). MAE values were moderately elevated compared to the original model publications, likely reflecting differences in sample characteristics, scanner specifications, and MRI acquisition protocols. The Brain Age Delta reveals a tendency among most models to underestimate brain age in our dataset, whereas CNN3 showed a profound overestimation (approximately 11 years). The age-bias slope quantifies the relationship between prediction error and chronological age, with values closer to zero indicating minimal age-dependent bias. All three models demonstrated moderate positive age-bias slopes, indicating that prediction errors increased with chronological age across models. These combined metrics provide an assessment of both the reproducibility and accuracy of the models in the context of spacefarer research. Notably, high reliability (ICC) does not guarantee validity, as demonstrated by CNN3’s excellent test-retest reliability despite poor predictive accuracy in our cohorts.

^*^The count before the slash represents the total number of scans, and the count after the slash signifies the number of repeated pairs analyzed for replicability assessment.

** Values originally reported as Median Absolute Error (MAE) accompanied by the standard deviation (SD) in the manuscript.

*** This denotes the MAE calculated across four distinct datasets: CamCAN, IXI, eNKI, and 1000-BRAINS, as originally documented in the study.

### Validation analysis

We evaluated model validity by comparing prediction performance (MAE / Median AE) against values reported in the original publications. All three models demonstrated higher MAE / Median AE in our dataset compared to their original reported values: MCCQRNN (Median AE: 3.33 y vs. 2.94 y), S4_R4 + GPR (MAE: 6.03 y vs. 4.04–5.20 y), and CNN3 (MAE: 11.1 y vs. 2.67 y). The moderately elevated MAE / Median AE values for MCCQRNN and S4_R4 + GPR likely reflect differences in sample characteristics, scanner specifications, and MRI protocols between our multi-site spacefarer cohorts and the original training datasets. Despite these differences, both models remained within acceptable prediction error ranges for estimating brain age. Model-specific validation revealed cohort-dependent performance variations. For MCCQRNN, the ESA control group showed the largest prediction error (Median AE: 4.96 y; 95% CI: 3.02–8.82), while for S4_R4 + GPR, ROS cosmonauts exhibited the largest deviations (MAE: 6.54 y; 95% CI: 5.70–7.26). Detailed validation metrics are provided in Table 1.

The CNN3 model, despite demonstrating excellent test-retest reliability (ICC: 0.97 in the pooled dataset), was excluded from subsequent analyses due to significant validity concerns. CNN3 exhibited substantial systematic bias with markedly elevated MAE, overestimating brain age by approximately 8 additional years beyond the expected range. Direct communication with the model developers revealed that CNN3 was trained predominantly on adults over 60 years old, creating a substantial training set mismatch with our younger spacefarer population (mean age: 44 y). While recalibration could potentially address this systematic bias and leverage CNN3’s robust cross-scanner reliability for pooled analyses, this was beyond the scope of the current study. Consequently, we proceeded with MCCQRNN and S4_R4 + GPR, both of which demonstrated adequate validity and excellent reliability in our dataset.

### Pre- and post-flight aging differences in Spacefarers

**Fig. 2:**
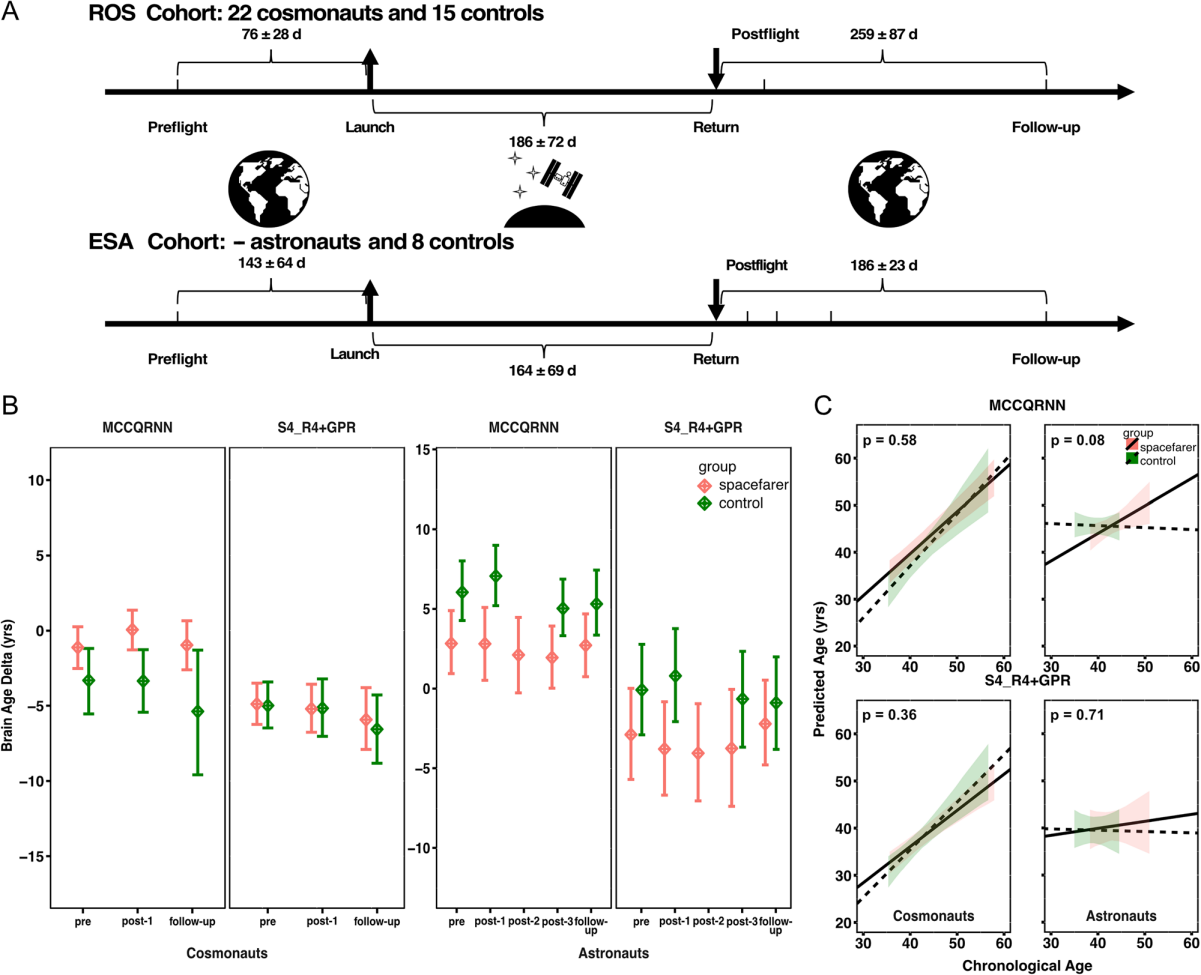
Comparative analysis of aging trajectories predicted by MCCQRNN and S4_R4 + GPR models. **A** This panel illustrates the composition of the study’s dataset, consisting of two distinct cohorts: ROS cosmonauts and ESA astronauts, who were enroled for the study at two sites over ten years. The interval regarding the data collection was reported as mean (SD). **B** Longitudinal brain age delta: This panel illustrates the mean brain age delta and its standard error across various time points for both cohorts, providing insight into the aging process at a discrete manner. **C** Upper panel, MCCQRNN Model Predictions: Displays a regression analysis of the predicted brain age versus chronological age using the MCCQRNN model, highlighting the different trend of Aging in the spacefarers and the controls, especially from the astronaut cohort. Lower panel, S4_R4 + GPR Model Predictions: Similarly, this panel presents a regression line for the predicted age against the chronological age derived from the S4_R4 + GPR model. They together offer a comparison of predictive performance between the two models. ROS cohort: cosmonauts (35.6–57.9 y) and controls (35.3–56.6 y); ESA cohort: astronauts (38.3–51.0 y) and controls (35.0–44.5 y).

A linear mixed model was fitted with brain age delta as the dependent variable over time points (sessions) and group, cosmonaut/astronaut vs. control subject, and individual subjects served as the random variable (See Table 2 for participant demographics and Fig. 2B for the longitudinal brain age trajectories). The MCCQRNN model showed a significant interaction between the group and session (F (2, 129.65) = 3.2556, *p* = 0.0417) in ROS cosmonauts. An exploratory post-hoc analysis showed that the difference in the pre-flight brain age delta is 0.842 years less than the immediate post-flight brain age delta after long-duration spaceflight, which means the prediction is 0.842 years younger at the pre-flight (estimate = –0.842, t = –2.31, *p* = 0.0225) in the ROS cosmonaut. Interestingly, controls were estimated albeit not significantly to be 2.985 years younger at post-flight than cosmonauts at post-flight (estimate = -2.985, t = –1.736, *p* = 0.0909). However, after FDR correction for 15 multiple comparisons, no pairwise contrasts reached statistical significance (all adjusted *p* ≥ 0.3375; see Supplementary Table 1 for complete statistics). These exploratory findings should be interpreted as hypothesis-generating signals requiring independent validation.

**Table 2:** Demographics of both LDSF cohorts

	Cosmonauts (*n* = 22)	ROS Controls (*n* = 15)	Astronauts (–)	ESA Controls (*n* = 8)
Age at pre-flight MRI scan (year)	43.5 (5.7)	42.8 (6.2)	–	39.5 (3.5)
Pre-flight MRI scan before launch (day)	75.7 (28.3)	–	142.6 (63.8)	–
Mission Duration (day)	185.6 (71.7)	–	–Similar to cosmonauts	–
Post-flight MRI scan after return (day)	9.1 (2.4)	–	4.0 (2.8)	–
Pre- and post-flight interval (day)	269.2 (80.3)	226.6 (66.8)	310.7 (82.8)	164.6 (39.1)
Follow-up MRI scan after return (day)	258.9 (86.5)	–	186.4 (22.5)	–
Pre-flight and follow-up interval (day)	527.5 (96.4)	495.8 (138.0)	493.1 (78.5)	362.5 (45.9)
Previous days in space (day)	108.7 (176.4)	–	–	–

Exact participant counts in the ESA cohort are suppressed to protect privacy due to the small sample size.

The S4_R4 + GPR model prediction did not show a significant interaction between session and group in the same statistical approach. The fewer number of subjects and the increased number of postflight time points for the ESA astronaut seemed to affect the linear mixed model analysis for both models. There was no significant interaction for the ESA cohort. Visual depiction of the brain age delta shows a U-shaped pattern for the ESA astronauts from preflight to postflight up until follow-up in both models alike (Fig. 2B right panel).

### Longitudinal aging trajectory

The longitudinally predicted brain age of both groups is shown in Fig. 2C. Predictions of the MCCQRNN model revealed that cosmonauts showed a less steep slope (ß = -0.15, indicating that for each additional year, cosmonauts’ predicted age decreased by 0.15 years relative to the control group, *p* = 0.5799), compared to the control group (β = 1.11, *p* < 0.0001). In the ESA cohort, astronauts exhibited a steeper brain age-chronological age slope compared to controls (group × age interaction: β = 0.63, *p* = 0.0770). This interaction coefficient indicates that the predicted brain age of astronauts increased by an additional 0.63 years per chronological year relative to controls. In contrast, the age of the control group did not show clear aging during this chronological period (β = –0.04, *p* = 0.8610).

Predictions from the S4_R4 + GPR model showed a similar pattern. In the ROS cohort, cosmonauts exhibited a slightly less steep brain age-chronological age slope compared to controls, though this difference was not statistically significant (group × age interaction: β = –0.24, *p* = 0.3642). Controls showed a slope of β = 1.01 (*p* < 0.0001), closely approximating the identity line, while cosmonauts’ slope was β = 0.77. The non-significant interaction indicates that age-related brain trajectories were similar between cosmonauts and controls in this cohort. In the ESA cohort, compared to the control group, spacefarers showed a numerically steeper slope in brain aging trajectory (group × age interaction: β = 0.18, *p* = 0.7120), whereas controls showed minimal change in predicted brain age (β = –0.03, *p* = 0.9360). This between-group difference did not reach statistical significance.

## Discussion

This exploratory study focused on investigating longitudinal brain age prediction as a novel quantification index derived from structural MRI images in space crew. Our preliminary results suggested a steeper aging trajectory in ESA astronauts compared to control participants, a trend observed in both MCCQRNN and S4_R4 + GPR models. Notably, the Brain age delta from ROS cosmonauts was found to be more pronounced in the post-flight group from the MCCQRNN model, in contrast to ESA astronauts, who exhibited an opposite pattern. Furthermore, differences in aging trajectories and changes pre- and post-flight were observed between the ROS and ESA cohorts. However, the interpretation of these findings remains uncertain—these changes may reflect either aging processes or reversible neurostructural adaptations that normalize post-flight, as suggested by the trend toward baseline in ESA follow-up data. A secondary aim of this study was to validate the employed machine learning models, i.e., MCCQRNN, S4_R4 + GPR, and CNN3. These models demonstrated good to excellent test-retest reliability. However, there was a noticeable increase in the MAE/median AE when compared to the validation MAE reported in the source studies. This might be partly due to the narrow age range of our cohorts (35–55 y) compared with the broader age range of MCCQRNN and S4_R4 + GPR.

Aging is a process characterized by the accumulation of structural and functional changes in an organism over time, resulting in the long run in a decline in physiological functions. Interestingly, physiological changes observed in astronauts after long-term spaceflight mirror those seen in the healthy aging population on Earth. A series of space studies have revealed that prolonged exposure to the space environment is associated with changes in several organ systems. These effects include decreased postural stability, sarcopenia, increased inflammation, and a notable rise in aging-related biomarkers^15–17^. Given that spacefarers possess a highly trained physiological system before space travel, they exhibit a resilience similar to that of healthy aging individuals on Earth, highlighting potential parallels between these two processes.

Focusing on the central nervous system, post-flight evaluations have uncovered extensive alterations in brain tissue. Notably, increased cerebrospinal fluid (CSF) volume has been reported across multiple cohorts^1,4,18^. Utilizing the same ROS cohort dataset as described in this paper, our research group has documented a pronounced reduction in grey matter (GM) volume within the orbitofrontal and temporal cortices, with a partial recovery to preflight levels after six months. Furthermore, we noted a global reduction in white matter (WM) and an expansion of perivascular spaces (PVS)^1,3,4,19^. In this study, we employed three ML models: MCCQRNN and S4_R4 + GPR, which use grey matter (GM) volume as predictor, offering a large data-based perspective to extend and enrich previous findings. The third model, CNN3, was designed to process the entire T1-weighted (T1w) MRI image. However, due to significant prediction errors encountered with our dataset, we were compelled to exclude CNN3 from our analysis.

Considering the specific characteristics of our dataset, which include its modest size and a few instances of missing data at follow-up sessions, we employed two complementary analytical approaches to evaluate the longitudinal data. One treats the measurement time point as a categorical variable (different sessions) to analyze the brain age delta pre, post-flight, and follow-up. This does not consider individual’s original age or the duration of the mission. Meanwhile, the other approach uses the original time scale as a continuous variable to examine brain age changes along a chronological timeline.

We predominantly focused on the ROS cohort for this analysis, as the limited sample size within the ESA cohort restricted our ability to achieve an adequate degree of statistical freedom. Our findings indicate a noteworthy trend in the cosmonaut’s group, where the postflight brain age predictions appeared marginally older when compared with the preflight brain age estimates derived from the MCCQRNN model.

Our cohorts are in their mid-adulthood, a phase characterized by gradual declines in brain structure and function^20^. Notably, our results revealed marked differences in brain aging trajectory in the ESA cohort between astronauts and controls (0.64 years older in astronauts compared with controls). This was consistently observed across two different models – MCCQRNN and S4_R4 + GPR – which both derive predictions from grey matter characteristics. The aging trajectory was also robust in the ROS cohort, while the ROS controls showed increased aging similar as cosmonauts. First, the congruence in findings suggests a robustness in the employed predictive approach. This is the first longitudinal age prediction study on the spacefarer cohort.

The aging trajectory was derived from individual spacefarers. Although cross-sectional studies associate elevated predicted brain age with adverse health outcomes, the interpretation of spaceflight-related increases remains ambiguous^21,22^. These changes may reflect either permanent aging processes or reversible neurostructural adaptations that normalize post-flight, as suggested by the trend toward baseline observed in the ESA cohort at follow-up (Fig. 2B). Going to space is challenging for the human body, as ample literature has reported the entirety of our physiological system changes^3,19,23^. Especially, Van Ombergen et al. have reported the GM and WM changes of ROS cohort in different brain regions after spaceflight^3^. The increased brain age delta in the postflight from ROS cosmonauts also suggests the GM alteration. Taken together our results from two analysis methods, it extended the understanding of brain physiology from a different perspective.

Our results show distinct patterns in aging trajectory and postflight brain age delta between ROS and ESA cohorts. These differences may be attributed to the implementation of varied countermeasures and exercise protocols. For example, ROS cosmonauts utilize lower body negative pressure as a countermeasure for LDSF, while ESA astronauts do not use this method. As Wuyts and colleagues also observed disparities between the ROS and ESA concerning perivascular space (PVS) enlargement^4^.

While assessing the reliability of our machine learning models, we observed several interesting patterns in their performance. The MCCQRNN model demonstrated high performance, the lowest Median AE, closely mirroring the findings of the original paper; we recorded a Median AE of 3.33, compared to the 2.94 reported originally. The S4_R4 + GPR model showed a mean MAE of 6.03 across the entire dataset, however, the CNN3 model predicted 11 years older for our dataset, this model supposed to have the lowest MAE among three models. Notably, a discrepancy became apparent when data was partitioned differently. For example, the largest Median AE from MCCQRNN model, observed in the ESA cohort control group, was 4.96. These variations suggest that while machine learning models may offer robust predictions at a population level, individual group characteristics can significantly influence outcomes. Dorfel et al. validated this model on another data set with a wide age range of 18 – 86 years and observed an MAE of 4.46^24^. Though we observed a trend in the longitudinal trajectory, we still may need future more robust and transparent normative brain age model to validate our hypothesis on brain aging in spacefarers.

The three models demonstrated good to excellent test-retest reliability across cohorts, as assessed by ICC, SEM, and MDC95 values^14^. For the pooled dataset, all models achieved excellent reliability (ICC > 0.90) with small measurement errors (SEM: 0.81–1.38; MDC95: 2.24–3.81). The CNN3 model consistently demonstrated the highest reliability across analyses, with ICC values ranging from 0.95 to 0.98 (SEM: 0.54–0.94; MDC95: 1.51–2.59) and the smallest measurement errors overall. However, high reliability reflects measurement consistency and reproducibility, not predictive validity. Model performance must be assessed in conjunction with outcome predictions, rather than relying solely on reliability metrics^25^. This distinction is particularly relevant for CNN3, which may require recalibration to improve predictive accuracy despite its superior reliability.

In summary, we found that while models like MCCQRNN and S4_R4 + GPR showed high performance, variations in MAE / Median AE were evident when analyzing different groups, suggesting that individual group characteristics significantly influence outcomes. Despite high test-retest reliability across all models, as indicated by excellent ICC values, the CNN3 model was excluded from the final analysis due to its disproportionately large MAE, highlighting the need for careful evaluation of model validity in addition to accuracy.

Our study’s key limitation was the narrow age range in our cohorts, with less than two years of variation individually and a 35–58 year range at the group level, which might limit the generalizability of our results. In our observations, both MCCQRNN and S4_R4 + GPR models exhibited similar aging patterns in the ESA cohort. However, to solidify these findings, further validation is essential, particularly across a broader range of ages in spacefarer cohorts. Additionally, the complexity of human aging, as outlined in the latest review by Walhovd et al., includes a list of factors such as early developmental stages and environmental influences that affect brain age^26^. Integrating a more extensive array of these factors from both the ESA and ROS cohorts into our models could significantly enhance their predictive accuracy and relevance, thereby providing a more comprehensive understanding of the brain aging process.

As an exploratory, methods-focused study, we utilized three machine learning models from different ML methodologies to predict brain age from spacefarers, demonstrating the feasibility of this approach. Our preliminary observations revealed potential aging trajectory differences in the ESA cohort, along with an increased post-flight brain age delta in ROS cosmonauts. Additionally, MCCQRNN and S4_R4 + GPR demonstrated good to excellent test-retest reliability and performed consistently with original publications, whereas CNN3 overestimated brain age by approximately 11 years. These findings provide a methodological foundation and preliminary insights into potential brain changes under LDSF. However, they underscore the necessity for extended long-term studies on human physiology under LDSF to confirm these exploratory findings and deepen our understanding.

## Methods

### Cohort demographics

This prospective cross-sectional study includes brain magnetic resonance imaging (MRI) data from two cohorts, ROS cosmonauts and ESA astronauts, along with respective control groups for each. This research is part of an ESA-endorsed prospective MRI study titled “BRAIN-DTI”. Cranial MRI scans were acquired in 22 male ROS cosmonauts and several ESA astronauts who embarked on long-duration missions to the ISS with a mean (SD) mission duration of 185.6 (71.7) days (ESA astronauts had a similar mission duration as ROS cosmonauts). We also collected brain MRIs from fifteen ROS and eight ESA healthy control participants. These controls were of similar age and education level as the space crews and were scanned in a comparable interval to account for aging effects. The detailed demographic and longitudinal time interval characteristics of the cohorts are reported in Table 2 and Fig. 2A.

The study was approved by the European Space Agency (ESA) medical board, by the Institutional Review Board of the Antwerp University Hospital (13/38/357), by the Committee of Biomedicine Ethics of the Institute of Biomedical Problems of the Russian Academy of Sciences, and the Human Research Multilateral Review Board (HRMRB). All participants, cosmonauts, astronauts, and healthy controls, provided written informed consent, and the investigations adhered to the principles outlined in the Declaration of Helsinki and its subsequent amendments. Due to privacy concerns from ESA management in light of the small cohort size, we only show group level data for this cohort.

### MRI protocol

The scientific MRI protocol included acquiring 3D high-resolution T1-weighted structural images (voxel size 1 × 1 × 1 mm). ROS cosmonauts and controls were scanned on a GE Discovery MR750 3 T MRI system (GE Healthcare, Milwaukee, Wisconsin) at the National Medical Research Treatment and Rehabilitation Centre of the Ministry of Health of Russia in Moscow, Russia equipped with a 16-channel receiver head coil with the following sequence parameters: T1-weighted fast spoiled gradient echo; 176 slices; TR = 7.9 ms; TE = 3.06 ms; TI = 450 ms; flip angle=12°. ESA astronauts and their respective control subjects were assessed on a dedicated 3 T MRI and PET scanner (Siemens mMR Biograph, Erlangen, Germany) located at the :envihab facility of the German Aerospace Centre in Cologne, Germany, using a 16-channel head and neck array coil. For each time point, two high-resolution sagittal T1-weighted 3D magnetizations prepared rapid gradient echo (MPRAGE) images were acquired approximately half an hour apart (TR 1900 ms; TE 2.43 ms; TI 900 ms; voxel size 1 × 1 × 1 mm; flip angle 9°; field of view (FOV) 256 mm, 176 slices; bandwidth 180 Hz/Px).

### Machine learning models

MCCQRNN model implements a Monte Carlo dropout composite quantile regression neural network. This model is specifically designed to adjust for uncertainties arising from noise in the data and the model itself. The package was trained on 10,691 subjects aged 20–72 years with a median absolute error (Median AE) of 2.94 years. Before running this model, raw T1w images were preprocessed using the default segmentation pipelines from the SPM12 toolbox CAT12 to extract the grey matter segmentation. MCCQRNN can be accessed via Docker or as a script (https://github.com/wwu-mmll/mccqrnn_docker)^11^.

S4_R4 + GPR (Gaussian process regression) stands out from 128 unique workflows derived from a combination of eight machine learning algorithms and sixteen feature representations. The training process of this model utilized four extensive neuroimaging databases, comprising 2953 images spanning adult ages from 18 to 88 years. It achieves a mean absolute error (MAE) of 4.73 years. The preprocessing protocol of S4_R4 + GPR included applying a smoothing kernel of 4 mm and resampling to a spatial resolution of 4 mm for the T1w images and then extracting the grey matter via CAT12 toolbox. Currently, the model has not been made open source^12^.

CNN3 is a modified 26-layer ResNet-based CNN using PyTorch, with 3D kernels and enhanced with data augmentation techniques such as rotations and gamma transformations. The model was trained on a large dataset comprising 17,296 T1w MRI images, with an age range of 32.0–95.7 years, and it demonstrated a mean absolute error (MAE) of 2.67 years. Streamlined with the raw T1w image as input, the fully trained CNN3 model is available on GitHub (https://github.com/westman-neuroimaging-group/brainage-prediction-mri)^13^.

### Statistical analysis

In this study, our longitudinal analysis was based exclusively on data from the first mission, whereas model validation used data from all missions. The ‘brain age delta’ was calculated by subtracting the chronological age from the predicted age.

Test-retest reliability was quantified using the intraclass correlation coefficients (ICC), specifically ICC(3,1) from the *psych* package v2.2.9 (two-way mixed-effects model, absolute agreement, single measurement), which treats subjects as random effects and measurement as fixed effects. ICC values were interpreted as: <0.50 = poor, 0.50 – 0.75 = moderate, 0.75 – 0.90 = good, >0.90 = excellent^14,27^. Standard error of measurement (SEM) was calculated as SEM = SD(differences)/ √2, where differences = Test – Retest. Minimal Detectable Change at 95% confidence (MDC95) was calculated as MDC95 = 1.96 × √2 × SEM, establishing the threshold for detecting true change with 95% confidence^28^.

Validity was assessed by comparing predicted brain ages to chronological ages. To account for within-subject dependence, all validity metrics were calculated at the subject level. For each subject, predicted ages were first averaged across test and retest scans, then brain age delta was calculated as Δ = Predicted Age – Chronological Age. Mean Absolute Error (MAE), MAE = mean(|Δ|), with 95% CIs from bias-corrected and accelerated bootstrap (1000 iterations), quantified the average error magnitude. Mean Error (ME), ME = mean(Δ), with 95% CIs from t-distribution, CI = ME ± t × (SD/√n). Median Absolute Error (Median AE), median(|Δ|), with IQR provided robust error estimates. Age-bias was assessed via regression (Δ ~ Chronological Age), where slope β₁ indicates the change in error per year (negative slopes: overestimation at young ages, underestimation at old ages).

We used the *lme4* package^29^ to perform linear mixed effects (LME) models for longitudinal analysis. Two a priori LME models were specified to test our primary hypotheses. The first LME model evaluated the relationship between brain age delta and categorical time points, with brain age delta as the outcome variable. Session (denoting longitudinal measurement points) and group were incorporated as fixed effects. The second LME model examined aging trajectories following spaceflight, using predicted brain age as the outcome variable with chronological age and group as fixed effects. For both models, the random effect structure included random slopes for chronological age and brain age delta within individual subjects to accommodate intra-subject variation. Statistical significance of fixed effects was evaluated using Wald tests via the *lmerTest* package.

Following the primary LME analyses, we conducted exploratory post-hoc pairwise comparisons using the *emmeans* package to extract estimated marginal means and perform pairwise contrasts across all group-session combinations^30^. Given the exploratory, hypothesis-generating nature of this study and the small sample size inherent to spacefarer research, we prioritized sensitivity for detecting potential patterns over strict Type I error control and therefore did not apply familywise error rate corrections^31,32^. Consequently, all post-hoc results should be interpreted cautiously as preliminary signals that require independent validation, rather than as confirmatory evidence. All analyses were conducted using R version 4.2.2 (R Core Team, 2022).

## Supplementary information

Supplementary_material

## Data Availability

The code used for analysis in this manuscript will be made available. However, due to privacy and ethical restrictions, the data itself will not be shared.
