# Supplementary material for: Longitudinal brain-age predictions comprising long-duration spaceflight missions
Source: NPJ Microgravity. 2026 Feb 18;12:24. doi: 10.1038/s41526-026-00575-3 (PMC12992904; doi:10.1038/s41526-026-00575-3)
Supplement: Supplementary file 1 — Supplementary_material [file 41526_2026_575_MOESM1_ESM.docx]

|  | contrast | estimate | SE | df | t.ratio | Uncorrected p | FDR-adjusted p |
| --- | --- | --- | --- | --- | --- | --- | --- |
| MCCQRNN | preflight control - followup control | -0.45 | 0.63 | 129.70 | -0.73 | 0.47 | 0.50 |
|  | preflight control - postflight1 control | 0.48 | 0.41 | 129.05 | 1.17 | 0.25 | 0.38 |
|  | preflight control - preflight cosmonaut | -1.66 | 1.72 | 37.02 | -0.97 | 0.34 | 0.42 |
|  | preflight control - followup cosmonaut | -2.20 | 1.73 | 38.38 | -1.27 | 0.21 | 0.38 |
|  | preflight control - postflight1 cosmonaut | -2.51 | 1.72 | 37.33 | -1.46 | 0.15 | 0.38 |
|  | followup control - postflight1 control | 0.93 | 0.63 | 129.76 | 1.49 | 0.14 | 0.38 |
|  | followup control - preflight cosmonaut | -1.21 | 1.78 | 42.56 | -0.68 | 0.50 | 0.50 |
|  | followup control - followup cosmonaut | -1.75 | 1.80 | 43.98 | -0.97 | 0.34 | 0.42 |
|  | followup control - postflight1 cosmonaut | -2.05 | 1.78 | 42.88 | -1.15 | 0.26 | 0.38 |
|  | postflight1 control - preflight cosmonaut | -2.14 | 1.72 | 36.93 | -1.25 | 0.22 | 0.38 |
|  | postflight1 control - followup cosmonaut | -2.68 | 1.73 | 38.29 | -1.55 | 0.13 | 0.38 |
|  | postflight1 control - postflight1 cosmonaut | -2.99 | 1.72 | 37.24 | -1.74 | 0.09 | 0.38 |
|  | preflight cosmonaut - followup cosmonaut | -0.54 | 0.43 | 130.60 | -1.26 | 0.21 | 0.38 |
|  | preflight cosmonaut - postflight1 cosmonaut | -0.84 | 0.36 | 129.96 | -2.31 | 0.02 | 0.34 |
|  | followup cosmonaut - postflight1 cosmonaut | -0.31 | 0.42 | 129.68 | -0.74 | 0.46 | 0.50 |
| S4_R4+GPR | preflight control - followup control | 0.03 | 0.75 | 130.16 | 0.04 | 0.97 | 1.00 |
|  | preflight control - postflight1 control | 0.60 | 0.49 | 129.08 | 1.22 | 0.23 | 1.00 |
|  | preflight control - preflight cosmonaut | 0.19 | 1.60 | 38.37 | 0.12 | 0.91 | 1.00 |
|  | preflight control - followup cosmonaut | 0.68 | 1.63 | 40.62 | 0.41 | 0.68 | 1.00 |
|  | preflight control - postflight1 cosmonaut | 0.59 | 1.61 | 38.88 | 0.37 | 0.71 | 1.00 |
|  | followup control - postflight1 control | 0.57 | 0.75 | 130.26 | 0.76 | 0.45 | 1.00 |
|  | followup control - preflight cosmonaut | 0.16 | 1.70 | 47.71 | 0.09 | 0.93 | 1.00 |
|  | followup control - followup cosmonaut | 0.65 | 1.72 | 50.09 | 0.38 | 0.71 | 1.00 |
|  | followup control - postflight1 cosmonaut | 0.57 | 1.70 | 48.24 | 0.33 | 0.74 | 1.00 |
|  | postflight1 control - preflight cosmonaut | -0.41 | 1.60 | 38.22 | -0.25 | 0.80 | 1.00 |
|  | postflight1 control - followup cosmonaut | 0.08 | 1.63 | 40.47 | 0.05 | 0.96 | 1.00 |
|  | postflight1 control - postflight1 cosmonaut | -0.00 | 1.61 | 38.72 | -0.00 | 1.00 | 1.00 |
|  | preflight cosmonaut - followup cosmonaut | 0.49 | 0.51 | 131.60 | 0.96 | 0.34 | 1.00 |
|  | preflight cosmonaut - postflight1 cosmonaut | 0.41 | 0.43 | 130.55 | 0.94 | 0.35 | 1.00 |
|  | followup cosmonaut - postflight1 cosmonaut | -0.08 | 0.50 | 130.12 | -0.16 | 0.87 | 1.00 |

Supplementary table 1: Complete exploratory post-hoc pairwise comparisons of brain age delta across group-session combinations from MCCQRNN and S4_R4+GPR.
